# The Germicidal Efficiency of Dental Cements

**Published:** 1915-04

**Authors:** Paul Poetschke

**Affiliations:** Experimental Chemist in the Caulk Laboratories


### THE GERMICIDAL EFFICIENCY OF DENTAL CEMENTS.

BY PAUL POETSCHKE,

EXPERIMENTAL CHEMIST IN THE CAULK LABORATORIES.

NOTE—This article is extracted from a paper published in *The Journal of Industrial and Engineering Chemistry*, for March, 1915, with permission of the author.

Dental cements, which are used because of their bactericidal properties, are the "Copper Cements" or "Copper Oxyphosphates." Their advantage over other dental cements lies in the increased germicidal power secured by the use of certain compounds of copper. There are several types of "copper cements." The powders appear as white, red, black, or varicolored compounds.

The liquid supplied with the majority of these cements consists essentially of concentrated solutions of orthophosphoric acid, $H_3PO_4$, modified by the addition of hydrated oxide of alumina, $Al_2(OH)_6$. Occasionally other metallic salts, such as iron and nickel, are added. Hydrated oxide of alumina is used as a modifier in order to control the reaction of the liquid on the powder when the two are combined by spatulation. White copper cement powder consists of calcined oxide of zinc, magnesia and bismuth, the copper being added in the form of cuprous iodide, $Cu_2I_2$, cupric phosphate, $CuHPO_4$, cupric silicate, $CuSiO_3$, or other light colored copper salts. Black copper cement powder consists of either black copper oxide, CuO, and cobalt oxide, or black copper oxide and zinc oxide with or without other oxides such as magnesia and bismuth. Red copper cement powder consists essentially of zinc oxide and cuprous oxide, $Cu_2O$, other oxides such as magnesia and bismuth being occasionally present. Red pigments are also added to improve the color, which is not a deception, provided sufficient cuprous oxide is used to give the cement mass the requisite germicidal power. The varicolored cement powders consist essentially of zinc oxide, magnesia, and bismuth, with a small proportion of light colored copper compounds such as cupric silicate, cupric phosphate, or other light colored copper salts. Pigments are added to obtain the various shades.

Very little information is contained in dental literature on the subject of germicidal efficiency of dental cements, and the little information which has been circulated is very often of a misleading character, having been presented largely for advertising purposes.

It is possible to measure the germicidal efficiency of material of this character in precisely the same way as it is possible to measure the germicidal efficiency of medicinal disinfectants. It is therefore obvious, especially in view of the varied composition of these cements, that a thorough investigation of this subject is of immediate interest to the dental profession, as well as to chemists and bacteriologists who may be called upon to report on the germicidal power of dental cements. The object of this paper is to show the comparative germicidal power of these cements, and also some of the compounds used in their production.

### EXPERIMENTAL—GERMICIDAL EFFICIENCY TESTS.

SERIES 1—CUPROUS OXIDE, CUPRIC OXIDE, AND CALCINED ZINC OXIDE—The object of these tests was to determine, if possible, the comparative germicidal efficiency of the red and black copper oxide, as compared with calcined zinc oxide.

Saliva was obtained from one individual by means of chewing unflavored gum.

Glass beads were added to 50cc. of saliva, in a sterile 4-oz. glass-stoppered bottle, and the bacteria separated by shaking for a period of ten minutes; 10 cc. of the saliva were transferred to each of five sterile 1-oz.glass-stoppered bottles; 1 cc. of the saliva was removed from each bottle, and the various dilutions plated in standard agar. One gram of each of the samples to be tested was added to the bottles in the order described below. The fifth bottle remained as a control on the number of bacteria present in the saliva during the test. All five bottles were shaken and then incubated at 37° C. for 24 hours. The bottles were then removed from the incubator and shaken thoroughly for one minute, and the sediment caused by the samples under examination was allowed to settle for five minutes. At the end of this time 1 cc. was removed from each of the five bottles and various dilutions plated in standard infusion agar. The plates in both series of determinations were incubated at 37° C. for a period of 48 hours, with the results given in Table I.

The results of these tests indicate that the amount of red copper oxide and black copper oxide which was dissolved by the saliva was sufficient to produce practical sterilization, and that the zinc oxide in itself, while not sufficient to produce practical sterilization, shows a germicidal efficiency of 96.2 per cent.

CUPROUS OXIDE, CUPRIC PHOSPHATE, AND CUPROUS IODIDE, IN ADMIXTURES WITH CALCINED ZINC OXIDE.

Series 2—The object of this test was to determine, if possible, the relative germicidal efficiency of cupric phosphate and cuprous iodide in comparison with cuprous oxide when these different compounds of copper were mixed with calcined oxide of zinc. The manner in which these tests were conducted was precisely the same as used in Series 1 and the results given in Table I show that the cuprous oxide and cuprous iodide in the quantities used, and in the presence of zinc oxide, produce practical sterilization in 24 hours. It is also shown that the cupric phosphate is less efficient under similar conditions than cuprous oxide or cuprous iodide.

Series 3—These tests were conducted with the object of determining still more definitely what the effect of cuprous oxide, cupric phosphate, and cuprous iodide would be in the presence of smaller quantities of these copper compounds in admixture with calcined zinc oxide. The tests were conducted in the same manner as in Series 2, except that 20 cc. of saliva were used for one gram of the powder, instead of 10 cc. for one gram, as in Series 2. The object of making this greater dilution was to reduce the quantity of powder in proportion to the saliva, so that the germicidal efficiency obtained in the different materials would be more pronounced.

The results indicate that these compounds of copper produce a marked effect in increasing the germicidal power. It will, however, be observed that the period of incubation of 24 hours is too long, or else that the dilutations are not sufficiently great to give definite information as to the germicidal efficiencies of the various compounds.

Table I—Tests of Copper Compounds and Zinc Oxide.

| | Per cent copper compound | Per cent calcined zinc oxide | No. Living Bacteria per Cc. Just before addition of 1 g. of sample | After incubation for 24 hrs. at 37° C. | Germicidal efficiency per cent |
|---|---|---|---|---|---|
| Series 1: | | | | | |
| Copper Oxide, red | 100 | None | 9,000,000 | Less than 100 | 99.9 |
| Copper Oxide, black | 100 | None | 8,200,000 | Less than 100 | 99.9 |
| None | | 100 | 10,500,000 | 394,000 | 96.2 |
| Control Saliva (Blank) | | | 9,400,000 | 12,900,000 | .. |
| Series 2: | | | | | |
| Cuprous Oxide | 1 | 99 | 8,800,000 | Less than 50 | 99.9 |
| | 5 | 95 | 9,500,000 | Less than 50 | 99.9 |
| | 10 | 90 | 8,400,000 | Less than 50 | 99.9 |
| Cupric Phosphate | 1 | 99 | 12,900,000 | 142,000 | 98.9 |
| | 5 | 95 | 10,300,000 | 200 | 99.9 |
| | 10 | 90 | 9,000,000 | 130 | 99.9 |
| Cuprous Iodide | 1 | 99 | 10,900,000 | Less than 50 | 99.9 |
| | 5 | 95 | 13,000,000 | Less than 50 | 99.9 |
| | 10 | 90 | 11,900,000 | Less than 50 | 99.9 |
| Control Saliva (Blank) | | | 10,700,000 | 8,900,000 | .. |
| Series 3: | | | | | |
| Cuprous Oxide | 0.1 | 99.9 | 5,600,000 | 550,000 | 90.1 |
| | 0.5 | 99.5 | 5,100,000 | 17,900 | 99.6 |
| | 1.0 | 99.0 | 5,500,000 | 100 | 99.9 |
| Cupric Phosphate | 0.1 | 99.9 | 7,400,000 | 12,600 | 99.8 |
| | 0.5 | 99.5 | 6,100,000 | 8,900 | 99.8 |
| | 0.1 | 99.0 | 6,200,000 | 41,000 | 99.3 |
| Cuprous Iodide | 0.1 | 99.9 | 5,900,000 | 335,000 | 94.3 |
| | 0.5 | 99.5 | 7,600,000 | Less than 100 | 99.9 |
| | 1.0 | 99.0 | 7,000,000 | 5,100 | 99.9 |
| Control Saliva (Blank) | | | 3,900,000 | 18,300,000 | .. |

The object of this work was largely to determine whether an increase in germicidal efficiency could be obtained with small quantities of copper compounds. The results of the tests, given in Table I, show without question that the various compounds of copper used in the tests show powerful germicidal action. It is, however, necessary to conduct these tests under the conditions in which the compounds will actually exist when used in a dental cement, since in the three series just described, the powders alone have been tested, and not the powders mixed into a cement mass with a balanced liquid and allowed to set.

For this reason the work on the uncombined powder was discontinued, and a further series of tests were conducted with the powder in combination with an appropriate liquid as a cement.

VARYING PERCENTAGES OF CUPROUS IODIDE ADDED TO A DENTAL CEMENT, COMPARED WITH TWO COPPER CEMENTS AND ONE CEMENT FREE FROM COPPER.

SERIES 4—The tests were conducted in the same manner as Series 3, except that the periods of exposure were 1 hour and 24 hours. The powders were prepared by combining the cement powders with the liquid, allowing the mass to set between glass plates for 48 hours, and then reducing the mass in an agate mortar, after which the powder was carefully sifted through a No. 25 bolting silk. The results are given in Table II.

They show that the various additions of cuprous iodide to Caulk's Crown and Bridge and Gold Inlay Cement produce complete sterilization in 1 and in 24 hours. It will be noted that Caulk's Crown and Bridge and Gold Inlay Cement, which does not carry any copper, is markedly germicidal. Ames' New Process Oxyphosphate of Copper also produces complete sterilization in 1 and in 24 hours. Smith's Copper Cement is shown to be deficient in germicidal power, since it fails to produce practical sterilization in 1 hour. An analysis of Smith's Copper Cement shows that it contains the equivalent of 0.755 per cent. copper oxide, CuO, which is apparently present either as copper hydrate or copper silicate. This quantity of copper and the nature of the compound is such that high germicidal power can not be obtained from the quantity which is used.

SERIES 5—This series is a duplication of Series 4, except that the exposure periods were reduced to 5 minutes, 15 minutes, and 1 hour, in order to more clearly show the germicidal efficiency, since, as observed in the case of Series 4, the various percentages of cuprous iodide did not show any difference, for the simple reason that the exposure period of 1 hour is sufficient to destroy all of the bacteria, whereas a shorter exposure period will not do so.

The results of Series 5 are given in Table II, where they are also expressed in terms of germicidal efficiency, which represents the percentage of organisms originally present which were killed by the cement.

A study of the data found in Series 5 shows that the addition of 1 to 5 per cent. of cuprous iodide to Caulk's Crown and Bridge and Gold Inlay Cement gives practical sterilization in five minutes exposure and complete sterilization in fifteen minutes exposure.

Ames' New Process Oxyphosphate of Copper apparently shows a higher efficiency when mixed with a nickel spatula than when mixed with an agate spatula. With the exception of the results obtained with this cement when mixed with an agate spatula, it is evident that practical sterilization is obtained in five minutes exposure and complete sterilization in fifteen minutes exposure.

Smith's Copper Cement shows incomplete sterilization throughout, and it is also seen that a slightly higher efficiency is obtained with this cement when mixed with a nickel, than when mixed with an agate spatula. This is true in both the five-minute and fifteen-minute exposure. There is apparently a slightly higher efficiency when mixed with an agate spatula in the one-hour exposure, but in this case the results are so closely identical that the conclusion appears warranted that this cement will give in precisely the same manner as with Ames' New Process Oxyphosphate of Copper, a slightly higher efficiency when mixed with a nickel spatula.

Caulk's Crown and Bridge and Gold Inlay Cement, while showing a slightly lower efficiency than Smith's Copper Cement in the five-minute and one-hour exposure, apparently shows approximately the same efficiency in the fifteen-minute exposure, and as far as the general results

Table II—Germicidal Efficiency Tests on Several Cements with Varying Periods of Exposure.

| SAMPLE | SERIES 4 No. OF LIVING BACTERIA PER Cc. Just before addition of 1 g. of sample | After exposure of 1 hr. | 24 hrs. | SERIES 5 No. OF LIVING BACTERIA PER Cc. Just before addition of 1 g. of sample | After exposure of 5 min. | 15 min. | 1 hr. | Per cent germicidal efficiency after exposure of 5 min. | 15 min. | 1 hr. |
|---|---|---|---|---|---|---|---|---|---|---|
| Series 4 and 5: | | | | | | | | | | |
| Ames' New Process Oxyphosphate of Copper: | | | | | | | | | | |
| Mixed with Nickel Spatula | 7,000,000 | 0 | 0 | 6,800,000 | 11,000 | 0 | 0 | 99.8 | 100 | 100 |
| Mixed with Agate Spatula | 9,000,000 | 0 | 0 | 5,000,000 | 210,000 | 100 | 0 | 95.8 | 99.9 | 100 |
| Smith's Copper Cement: | | | | | | | | | | |
| Mixed with Nickel Spatula | 15,000,000 | 2,000,000 | 0 | 7,900,000 | 3,400,000 | 3,000,000 | 1,000,000 | 56.9 | 62.0 | 87.3 |
| Mixed with Agate Spatula | 15,000,000 | 770,000 | 0 | 5,300,000 | 3,400,000 | 2,800,000 | 500,000 | 35.8 | 47.1 | 90.5 |
| Caulk's Crown & Bridge & Gold Inlay Cement Containing Per cent Cuprous Iodide: | | | | | | | | | | |
| 1 .................... | 14,000,000 | 0 | 0 | 5,200,000 | 143,000 | 0 | 0 | 95.3 | 100 | 100 |
| 2 .................... | 10,000,000 | 0 | 0 | 6,000,000 | 39,000 | 0 | 0 | 99.4 | 100 | 100 |
| 3 .................... | 7,400,000 | 0 | 0 | 5,000,000 | 2,500 | 0 | 0 | 99.9 | 100 | 100 |
| 4 .................... | 8,900,000 | 0 | 0 | 8,000,000 | 200 | 0 | 0 | 99.9 | 100 | 100 |
| 5 .................... | 12,000,000 | 1,800 | 0 | 10,000,000 | 2,500 | 0 | 0 | 99.9 | 100 | 100 |
| 0 .................... | 11,000,00 | 4,500,000 | 0 | 7,000,000 | 5,000,000 | 3,800,000 | 2,000,000 | 28.5 | 45.7 | 71.4 |
| Control Saliva (Blank)... | 12,000,000 | 10,000,000 | 17,400,000 | 6,000,000 | 8,000,000 | 7,000,000 | 8,000,000 | .. | .. | .. |
| Series 6: | | | | Series 6 | | | | | | |
| Smith's White Copper Cement.......................... | | | | 9,000,000 | 11,000,000 | 3,000,000 | 1,500,000 | 0 | 66.6 | 83.3 |
| Caulk's White Copper Cement.......................... | | | | 10,000,000 | 360,000 | 100 | 0 | 96.3 | 99.9 | 100 |
| Caulk's Red Copper Cement............................ | | | | 7,000,000 | 200,000 | 0 | 0 | 97.1 | 100 | 100 |
| Ames' New Process Oxyphosphate of Copper Cement... | | | | 10,000,000 | 400,000 | 0 | 0 | 96.0 | 100 | 100 |
| Decido Copper Cement................................. | | | | 10,000,000 | 9,000,000 | 400,000 | 200,000 | 10.0 | 96.0 | 98.0 |
| Fellowship Oxyphosphate of Copper Cement............ | | | | 7,000,000 | 7,000,000 | 250,000 | 200,000 | 0 | 96.4 | 97.1 |

show there is but little difference between this cement and Smith's Copper Cement, as far as the germicidal efficiency is concerned. It must be remembered, however, that Caulk's Crown and Bridge and Gold Inlay Cement is not a copper cement, and therefore it will be seen that Smith's Copper Cement is deficient in the germicidal power to be expected of a copper cement.

### SERIES OF COPPER CEMENTS NOW ON THE MARKET.

Series 6—These tests were conducted with the cement mixed in all cases by means of an agate spatula. In other respects the technique was the same as that employed in the tests in Series 5. The results, given in Table II, show that Caulk's White Copper Cement, Caulk's Red Copper Cement, and Ames' New Process Oxyphosphate of Copper Cement are almost identical in germicidal power. Decido Copper Cement and Fellowship Oxyphosphate of Copper Cement require fifteen minutes exposure in which to secure the germicidal efficiency obtained from Caulk's White Copper Cement, Caulk's Red Copper Cement, and Ames' New Process Oxyphosphate of Copper Cement in only five-minutes exposure. This shows that Decido Copper Cement and Fellowship Oxyphosphate of Copper Cement are both much weaker in germicidal power than these cements. Smith's White Copper Cement shows the lowest germicidal power of the entire series.

### TESTS OF THREE CEMENTS AND ONE MIXTURE.

Series 7—Caulk's Copr-Zinc is a preparation containing ten per cent. cuprous iodide, and therefore has five times the concentration of Caulk's White Copper Cement. It is not used alone as a cement, but is used in the proportion of one part of Copr-Zinc to four parts of Crown and Bridge and Gold Inlay Cement.

The technique followed in Series 7 varied slightly from that followed in the other tests, particularly in that only fifteen-minute exposures were made, and that the powder and saliva were thoroughly shaken immediately before plating. Only one dilution was plated, representing 1-10,000 cc. of the original saliva. Individual controls were eliminated, one control serving for this series. The photographs show very plainly the growth of colonies in the saliva itself, as well as in the saliva exposed to Caulk's Crown and Bridge and Gold Inlay Cement and Smith's Copper Cement. It was, however, observed that in the case of Caulk's Crown and Bridge and Gold Inlay Cement to which Copr-Zinc was added, and Ames' New Process Oxyphosphate of Copper Cement, the plates were both sterile.

### METHODS IN USE FOR DETERMINING THE BACTERICIDAL PROPERTIES OF DENTAL CEMENTS.

The experimental part of this paper clearly shows that the germicidal power of a dental cement can be determined. It is evident that a standard or uniform method of testing should be employed in work of this character, and unless the technique is given, it is obvious that such work would be of little value.

### AUTHOR'S METHOD.

The following method has been developed as a result of the experimental work given in this paper:

Preparation of the cement—The manufacturers of dental cements almost invariably give the instructions to be followed in the mixing of a given cement, and therefore it is only fair that these instructions be rigidly observed. The cement is therefore mixed in accordance with the instructions, using whatever character of spatula is recommended, and the cement mass placed between glass plates, separated by glass guides so as to form a disk of the cement. It is then allowed to set for a period of 48 hours, after which it is powdered in an agate mortar and carefully bolted through No. 25 bolting silk. Metallic sieves should never be used for the purpose. A sufficient number of mixes are made to yield approximately 3 g. of powder. This powder is placed in a glass vial and is now ready for the test for germicidal efficiency.

SALIVA is obtained from one or more individuals by means of chewing paraffin. The individual is instructed to rinse the mouth with water, and then to chew a piece of paraffin, rejecting the sailva until the mass of paraffin is soft and adherent, after which the saliva is collected in a sterile glass-stoppered bottle until a sufficient quantity has been obtained. If the saliva from more than one individual is collected, it is all finally mixed and strained through several thicknesses of sterile cheese-cloth, in order to remove large particles from the saliva. Sterile glass beads are then added to the strained saliva and the bacteria separated by shaking vigorously.

TECHNIQUE—Twenty cc. of saliva are measured into a sterile 1-oz. glass-stoppered bottle and the bottle placed in a water bath maintained at a temperature of 37° C. One gram of the prepared cement powder is weighed on a clock glass, and held in readiness for its addition to the saliva. The bottle containing the saliva is then shaken for one minute, 1 cc. withdrawn and plated in the proper dilution as an individual control. One gram of powder is then added to the bottle, the bottle thoroughly shaken for one minute, and transfers made at intervals of five and fifteen minutes, proper dilutions of these transfers being plated. During the exposure period, the bottles are shaken for a minute at intervals of five minutes, the final shaking of one minute being made before the final transfer. The proper dilution is then plated in nutrient agar-agar and incubated at 37° C. for 48 hours. If more than one test is to be conducted at one time, it is simply necessary to repeat the above procedure. Experience with this method has shown that dilutions of 1-10,000 cc. invariably yield plates which can be properly counted, although in some instances a dilution of 1-100,000 cc. is preferable in the case of the saliva itself.

NOTES—The object of using a hardened cement, one in which the chemical reactions involved have reached equilibrium, is that in this condition all of the cements to be compared have reached a fixed state. The results are not influenced by such factors as the setting time. A cement which could only act germicidally in a plastic condition, loses this property on setting, and would obviously be of no permanent value. Any liquid disinfectant applied to the cavity before inserting the cement would do as much work. It is the continued giving off of germicidal salts after the cement mass has set, which is the important factor. This is accomplished through the gradual yet infinitesimal solubility of the cement. A cement which is germicidal in the hardened state will certainly be germicidal in the plastic state, if not more so. From a practical standpoint, it would be impossible to determine germicidal efficiency accurately by using a cement in the plastic state. *First:* It would be almost impossible to use a fixed and uniform amount of such a plastic in all of the tests; any variation in the amount of the plastic would introduce a variable. *Second:* It would be practically impossible to control the state of the mix, since this is continually varying while in the plastic state, and therefore, another variable would be introduced. It is necessary to work with fixed conditions in order to obtain comparative results.

The object of using the cement in the powdered condition in which the particles of powder are of as uniform size as it is possible to obtain them by careful sifting, is that a given weight of such powder will represent a fairly uniform surface area of the cement. Since all of the cements in the tests are prepared in exactly the same manner, it is evident that this introduces a fixed condition.

Saliva is used in the tests, since in this manner the various mouth organisms are used instead of a pure culture of one organism. Saliva is the proper media to be employed in tests of this kind, since it represents exactly the fluid with which the cement comes in contact in the mouth. The exposure of masses of cement to ordinary culture media does not represent the conditions to be obtained in the mouth.

It will be remembered that culture media is always acid in reaction, whereas the saliva is normally slightly alkaline. Bacteriologists adjust the acidity of culture media to a reaction of "(+1)" a symbol used to indicate that one per cent. of normal sodium hydroxide solution is required to bring it to the phenolphthalein neutral point. If saliva is tested in accordance with the standard method adopted by bacteriologists for ascertaining the reaction of culture media, it will be found that the saliva will not be acid to phenolphthalein, but alkaline. It is thus evident that saliva is the proper media to be used for tests of this character, since all tests where nutrient agar-agar, or nutrient bouillon is used, must of necessity fail to represent the conditions which exist in the mouth, because the reaction and chemical composition of saliva and culture media are entirely different.

The importance of shaking the saliva and the cement powder immediately before withdrawing a sample for plating will be realized when we consider that if the sample is merely allowed to settle, the cement powder, in settling, may carry down with it some living bacteria, or else the bacteria may be unequally distributed.

### OTHER METHODS.

The following statements bearing directly on the subject of germicidal properties are made by W. V-B. Ames in the *Dental Review*, June, 1914:

1—''The results obtained after placing hardened cement pellets in media are apt to be misleading, because the greater the density and integrity of the mass, the less readily will germicidal salts be given off.

2—''In a liquid medium such as beef bouillon, a tendency to form a copper salt distinctly blue is easily seen when a pellet of a high percentage of copper oxide cement is placed in sterile medium, with no visible tendency, when a pellet containing, for instance, fifty per cent. each of copper and zinc oxide modified with smaller additions of any copper salt is so placed. In a tube of inoculated bouillon the same blue color will appear and if extreme care is exercised to avoid agitation of the fluid medium, there will be inhibition about the pellet at the bottom of the test tube. If only a low percentage of copper compound be contained in the pellet, no inhibition or color will be noticeable.''

3—''Pellets of cement subjected to various media, both sterile and inoculated, give evidence of their comparative germicidal potency. When bacteria will thrive throughout an amount of liquid media containing pellets of zinc oxide cements, pellets compounded of 25 per cent. to 75 per cent. of copper oxide with zinc oxide, with pellets of some so-called copper cements composed of zinc oxide and a small percentage of iodid of copper, and even pellets of oxychlorid of zinc, and when the media in the test tube to the level of the top of a pellet of a high per cent. copper oxide cement will remain sterile and clear excepting a copper salt color, we have evidence of comparative germicidal properties.''

4—''Bouillon experiments are otherwise very unsatisfactory, as the conditions differ entirely from those we are trying to explain. Agar as media offer slightly better opportunity to obtain tooth and filling conditions. Various schemes by which pellets and agar have been inoculated and brought into contiguity, have been tried. The method most nearly duplicating the tooth and filling condition has been the filling of the smallest Wassermann test tube with melted and inoculated agar, to within about one-eighth of an inch of the top and chilling the tube and contents. While this agar is yet cold, the cement in question is inserted in a plastic state, to fill the remaining portion of the tube. A mass of cement placed upon inoculated agar in a Petri dish does not as closely duplicate the tooth and filling conditions, but the comparative inhibition is more apparent.''

Quotation one shows that Ames is opposed to testing a cement in the hardened state. It must, however, be remembered that a cement inserted into the tooth cavity hardens in a very short time; hence, if the mass is not germicidal in the hardened state, it will fail to accomplish the purpose for which it is used. A cement which could act germicidally only in a plastic condition would lose this property in setting, and obviously be of no permanent value, since any disinfectant applied to the cavity before inserting the cement will do as much work. The cement remains in the plastic condition in the tooth cavity for a very short time, whereas it remains there in a hardened condition for a period of years, and it follows that the proper condition in which the cement should be tested is in the hardened state, and not in the plastic form, since the dentist does not rely upon the initial sterilization secured by inserting the plastic mass into the cavity, but upon the permanent sterilization secured by the gradual, although infinitesimal solubility of the hardened cement mass.

The first part of Quotation 2 is intended to show that in the nutrient beef bouillon, hardened pellets of cement composed of a high percentage of copper oxide, when placed in the sterile bouillon, will form a distinctly blue color in the bouillon, due to the solution of copper salts, whereas a pellet composed of fifty per cent. each of copper oxide and zinc oxide modified with smaller additions of any copper salt, fail to show this blue color. This is simply a function of the solubility of the cement, and if anything, it merely shows that a high percentage of copper oxide cement is less resistant, or more soluble in nutrient bouillon, than one composed of a smaller percentage of copper oxide, and admixed with zinc oxide. The second part of this quotation is intended to show that, owing to the presence of this blue copper salt, there will be inhibition of bacterial growth about the pellet at the bottom of the test tube, but that with a low percentage of a copper compound, no inhibition or color will be noticeable. It would certainly be necessary to exercise extreme care to avoid agitation of the nutrient bouillon, if this inhibition is to be observed, but the important point is that even if it were possible to make this observation, the test does not show the germicidal efficiency of the material. Again, the chemical composition of nutrient bouillon is entirely different from that of the saliva, so that a test of this character has no value.

Quotation 3 infers that the comparative germicidal potency of various copper cements can be determined by placing hardened pellets of the cement mass in nutrient bouillon by observing whether bacteria will thrive throughout the liquid media. It is inferred in this quotation that those copper cements composed of copper oxide and zinc oxide, and also those composed of zinc oxide with a small amount of cuprous iodide, and even oxychloride of zinc cements, show no germicidal power, and that only those cements which contain a high percentage of copper oxide have germicidal power, because the media "will remain sterile and clear, excepting a copper salt color." The experimental part of this paper clearly shows how absurd this statement is, since it is shown conclusively that those materials which Ames infers to have no germicidal power, on the contrary, have very high germicidal efficiency—as great as can be obtained with cements composed largely of copper oxide. Assuming for the moment that the statement is based upon accurate observation, it is evident that the test is of a qualitative character, giving no information whatever as to germicidal efficiency.

Quotation 4 also fails to present a method which would be of any value in determining germicidal efficiency. The assumption that the filling of the smallest Wassermann test tube with melted and inoculated agar will most nearly duplicate the tooth and the filling condition, is certainly not impressive, mainly on account of the fact that agar is entirely different in physical and chemical character from the tooth substance, and the mere crowding of a mass of cement into a space approximately the size of a tooth cavity does not duplicate the tooth and the filling conditions. Here again the tests used are based upon the observation of comparative inhibition of bacterial growth, and not upon the determination of the percentage of organisms destroyed in exposure, and for this reason the tests fail to give any information on the germicidal efficiency of the materials.

In addition to the above statements, Ames shows two plates in which were embedded freshly mixed cement upon agar inoculated with staphylococcus. In explaining these plates, Ames makes the following statement:

"In applying these cements in the fresh state, to inoculated agar, there is one case, No. 1, the typical copper coloration with marked inhibition of bacterial reproduction and in the other, No. 3, no coloration and inhibition, only to the extent that would be expected from the corresponding zinc salts, which we know from clinical observation is so slight as to be negligible."

No. 1 refers to a copper cement composed largely of copper oxide, and No. 3 refers to a white copper cement. It will be noted that Ames claims that the white copper cement gave "inhibition only to the extent that would be expected from the corresponding zinc salts." The experimental part of this paper shows that there is a vast difference between a white copper cement and one which is free from copper, and the very fact that Ames is forced to use the expression, "only to the extent that would be expected from the corresponding zinc salts," shows that the test is of purely qualitative character and gives no information as to germicidal efficiency. There is a vast difference between an ordinary oxyphosphate of zinc cement and an oxyphosphate of zinc cement containing cuprous iodide. What does "inhibition only to the extent to be expected from corresponding zinc salts" mean in the absence of a definite statement as to what the zinc salts will accomplish? We must have some definite standard of comparison.

To test the antiseptic properties of a plastic and a set cement mass two inoculated Petri plates were used, in one a soft or plastic mix was placed and in the other a set mass.

A Petri dish containing agar inoculated with saliva upon which two mixes of plastic masses of cement were placed was incubated for 48 hours. Caulk's Crown and Bridge and Gold Inlay Cement and Caulk's Crown and Bridge and Gold Inlay Cement and Caulk's Copr-Zinc (four parts of the former to one part of the latter, by weight) were used. Colonies of bacteria were seen over the entire plate. There was a sterile area around each cement mix, the actual measurement on the original plate showing sterility within the radius of from six to eight mm. in both cases. It would have been impossible to specify the comparative germicidal properties of these two cements from this test, since it failed to show any marked difference in the germicidal power. Reference to the experiment shown in Series 7 of the experimental part of this paper plainly shows that the same materials vary widely in germicidal power.

Another Petri dish containing agar inoculated with saliva into which two solid cylinders of the same cement were inserted shows the bacterial colonies to approach the cement mass much more closely than in the case of the plastic mass. This is exactly what one would expect, owing to the insolubility of the set cement as compared with the solubility of the unset or plastic mass. Here again it would have been impossible to state whether one cement was more germicidal than the other for the same reason above stated.

### SUMMARY.

Dental cements known as "copper cements" vary widely in chemical composition. The several types sold to the dental profession are at present described almost entirely by the color of the powder, and include red, white, black, and varicolored cements. The color of these cements is largely due to the compounds of copper used in their production, although pigments are used to improve the color. Such additions may be considered to be legitimate if the cement shows the requisite germicidal power.

Cuprous oxide, cupric oxide, and zinc oxide have marked germicidal properties. The addition of cuprous oxide, cupric phosphate, and cuprous iodide, to zinc oxide, enhances the bactericidal properties of the zinc oxide.

The addition of varying amounts of cuprous iodide to a "copper-free" dental cement shows that the germicidal efficiency is increased in proportion to the quantity added. The addition of one per cent. of cuprous iodide to a dental cement increases considerably the germicidal efficiency.

Commercial "copper cements" show wide differences in germicidal efficiency, the color of the cement having no relation to its bactericidal properties.

Tests for germicidal properties of dental cements in "plastic" or "set" condition are unsatisfactory if such mixes are placed in inoculated bouillon or agar-agar, owing to the difference in chemical composition of the nutrient culture media and the saliva. Visual tests for inhibition of bacterial growth are purely qualitative tests and fail to demonstrate the comparative germicidal efficiency of these materials. The comparative germicidal efficiency of "copper cements" can be ascertained only by methods which determine the number of living organisms killed on exposure to the cement under fixed conditions.

